# The Concordance between Myocardial Perfusion Imaging and Coronary Angiography in Detecting Coronary Artery Disease: A Retrospective Study in a Tertiary Cardiac Center at King Abdullah Medical City

**DOI:** 10.1155/2016/9847575

**Published:** 2016-06-27

**Authors:** Fatma Aboul-Enein, Majed O. Aljuaid, Hail T. Alharthi, Abdulkarim M. Almudhhi, Mohammad A. Alzahrani

**Affiliations:** ^1^Department of Adult Cardiology, KAMC, Makkah 21955, Saudi Arabia; ^2^Medical College, Taif University, Taif 21974, Saudi Arabia

## Abstract

*Background*. Coronary artery disease (CAD) is considered as the leading cause of the cardiovascular fatalities worldwide. CAD is diagnosed by many modalities of imaging such as myocardial perfusion imaging (MPI) and coronary angiography (CAG).* Methods*. A retrospective cross-sectional study was conducted that included all patients referred to the KAMC (King Abdullah Medical City) nuclear cardiology lab from its opening until the end of May 2014 (a period of 17 months). A total of 228 patient reports with a history of conducting either CAG or MPI or both were used in this study and statistically analyzed.* Results*. An analysis of the MPI results revealed that 78.5% of the samples were abnormal. On the other hand, 26.75% of the samples revealed that they were subjected to CAG and MPI. There was a significant and fair agreement between MPI and CAG by using all the agreement coefficients (kappa = 0.237, phi = 0.310, and *P* value = 0.043). The sensitivity, specificity, and accuracy of MPI with reference to CAG were 97.8%, 20%, and 78.69%, respectively. In addition, positive predictive and negative predictive values were 78.95% and 75%, respectively.* Conclusion*. In a tertiary referral center, there was a significant agreement between MPI and CAG and a high accuracy of MPI. MPI was a noninvasive diagnostic test that could be used as a gatekeeper for CAG.

## 1. Background

Cardiovascular diseases (CVDs), especially the coronary artery diseases (CADs), are among the leading causes of fatalities worldwide [1–3]. CAD caused more than 7 million deaths worldwide in 2001 [2]. It causes more than 4.5 million deaths in the developing countries [3]. About 5.5% of the population in Saudi Arabia is suffering from these diseases [4]. CAD is diagnosed by many modalities of imaging. Although coronary angiography (CAG) is invasive, it is considered as the gold standard for CAD diagnosis [5].

CAG is used to show the patency of the coronary arteries by using a contrast medium and radiographic visualization [6]. Many complications have limited its use, including arrhythmia, aneurysms, arteriovenous fistulas, hemorrhage and hematomas, perforation of the heart or great vessels, allergic reactions, embolisms, infections, and death [6]. One of these modalities that are used to detect CAD other than CAG is myocardial perfusion imaging (MPI), which is a widely available noninvasive test that is indirectly showing how well blood reaches the myocardium by using radiopharmacological agents [7]. Single photon emission computed tomography (SPECT) and positron emission tomography (PET) are the two techniques used for MPI [7]. SPECT was used at the hospital in which this study was done.

In terms of indications and contraindications of MPI and CAG comparatively, CAG is more dangerous compared to MPI. CAG has indications in many cases such as unstable angina, chronic stable angina, and coronary syndrome and when used before a bypass surgery, while MPI is used in the evaluation of myocardial perfusion abnormalities in patients with a low to moderate likelihood of CAD as well as when suggested by the location, extent, and severity of chest pain [6, 7]. CAG is contraindicated in multiple conditions including allergy to dye, hypertension, coagulopathy, and kidney failure. On the other hand, MPI is not used in many cases like recent myocardial infarction, the inability to fulfill the exercise stress test fitness criteria, and contraindication conditions to adenosine stress testing [6, 7].

Patients with chest pain, especially when it is atypical and they have low to intermediate likelihood of CAD, need to show objective evidence of ischemia (ECG/MPI) before being referred for CAG with consideration that exercise ECG had no added prognostic value in the presence of normal findings in stress MPI [8]. Two meta-analyses involving a total of 6972 patients had been conducted to calculate the sensitivity and specificity of MPI in detecting CAD with reference to echocardiography, which found that sensitivity and specificity were ≥87% and ≥73%, respectively [9]. On the other hand, sensitivity and specificity of MPI with reference to CAG for 96 patients were 95% and 83%, respectively [10]. Patients with syndrome X and the perfusion defect with normal coronary angiography were found to be at risk for the development of coronary event (acute coronary syndrome) [11]. Similarly, patients with abnormal MPI and normal CAG were more liable to develop CAD, especially if CAG was after revascularization, as the results of both investigations in this phase will vary throughout this period [12].

MPI is used for detecting ischemia in the myocardium. The utility of the MPI in the CAD is a controversial point from many perspectives such as priority and dependence in the clinical decision. These perspectives had been argued by many researchers who had studied and weighed the anticipated high accuracy, specificity, and sensitivity against the projected risks and as consequences of dependence on its results in a clinical scenario [13–16].

The aim of the current study was to evaluate the relationship between MPI and CAG in a tertiary cardiac center at King Abdullah Medical City (KAMC).

## 2. Materials and Methods

The current study is a retrospective cross-sectional study that included all patients who were referred to the KAMC (King Abdullah Medical City) nuclear cardiology lab and have available reports from its opening until the end of May 2014 (a period of 17 months). Data was collected from patients' electronic health records and placed into an Excel sheet using a hospital computer in the department, not showing any nominative information. The patients were identified by a serial study code and their initials. These were linked to patients' names and their medical record number (MRN) in a separate identification log sheet, which had been kept in a safe locked place. After verification, data were transferred to the statistical database directly by using SPSS. The reports of MPI and CAG performed for those patients were examined. Files of patients with negative MPI reports were reviewed for possible subsequent referral to CAG 3 months after receiving a negative MPI to ensure that all the cases where patients took both tests were included in this study in hope to minimize the effect of the referral bias. The duration of follow-up was the mean of interval periods that separate CAG and MPI among patients of KAMC. Since CAG is considered the gold standard for CAD, patients who have been subjected to both tests are statistically investigated. The CAG has to be subsequent to MPI in order to be involved in this study. The IRB of KAMC had approved the waiver of the informed consent as it was a retrospective study.

### 2.1. Coronary Angiography Protocol

The standard Judkins approach was used in performing the coronary angiography. The angiograms were analyzed specifically for this study by one observer who was unaware of the clinical and scintigraphic data.

### 2.2. Rest-Stress MPI Protocol

Stress/rest separate acquisition 99mTc-sestamibi MPI was used [17]. Patients who used agents which affected the stress study were instructed to discontinue their use before the stress test was performed such as the consumption of antihypertensives, nitrates, and caffeine products. There are two major types of stress tests that are pharmacological (85.5%) and use coronary vasodilators such as adenosine and exercise stress tests using the Bruce protocol (14.5%). One injection of 99mTc sestamibi at peak stress was given to patients who were subjected to exercise stress [17]. Exercise at high speed and grade was continued for 1 min after injection and for an extra 2 min at lower speed and grade [17]. For adenosine stress, adenosine was given intravenously at a dose of 140 g/kg/min for 6 min. At the end of the second or third minute of infusion 99mTc sestamibi was injected, and approximately 1 h later SPECT was conducted [17]. These two stress protocols are used according to the patient's characteristics; for instance, if the patient was young and had no previous MI, physician could use an exercise protocol, while in the elderly and patients with previous MI he/she must use the pharmacological method [7]. On a separate day, another dose of 99mTc-sestamibi was given at rest and a patient's image was taken after 1 hour.

### 2.3. MPI Acquisition Protocol

Acquisition was done using Siemens Symbia T-16 SPECT-CT, dual head gamma camera, using standard energy Windows for Tc-99 sestamibi [17]. Image analysis was done using Syngo MI software and a 4 DMSPECT package.

### 2.4. Image Analysis

**Figure 1:**
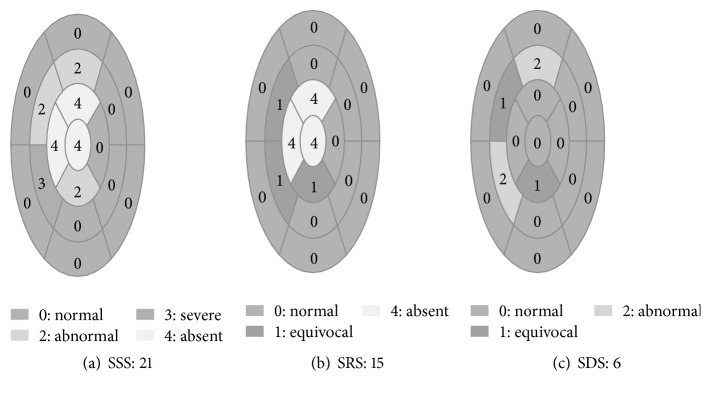
The 17-segment model with a 5-point scoring system for MPI. Circles (a) and (b) show the MPI images during the stress test and rest, respectively. Circle (c) is the difference between (a) and (b) MPI images. SSS: summed stress score; SRS: summed rest score; SDS: summed difference score.

The 17-segment model of MPI images was used for scoring. The 5-point scoring system was used to assess each segment: 0: normal; 1: equivocal; 2: moderate; 3: severe reduction of radioisotope uptake; and 4: absence of detectable tracer uptake in a segment (Figure 1). Summed stress score (SSS) is calculated by adding the 17 segment scores of the stress images while summed rest score (SRS) was calculated by adding the 17 segment scores for rest images. Summed difference score (SDS), the difference between stress and rest scores, is measuring the defect induced by stress (Figure 1). MPI results were considered normal if SSS < 3 and abnormal if SSS ≥ 3 [18].

### 2.5. Statistical Analysis

Patients who were subjected to MPI and CAG were classified as either an ischemic group or nonischemic group (Table 2). The ischemic group included true positive “TP” and false negative “FN.” TP is defined as the patient who was classified as positive in both tests. Also, FN is defined as the patient who was classified as negative by MPI while being classified as positive by CAG. On the other hand, the nonischemic group included the true negative “TN” and the false positive “FP.” TN is defined as the patient who was classified as negative in both tests. FP is defined as the patient who was classified as positive by MPI while being classified negative by CAG. MPI's sensitivity, specificity, accuracy, positive predictive value, and negative predictive value were calculated as described by the Altman method which is as follows: sensitivity = TP/(TP + FN), specificity = TN/(FP + TN), accuracy = (TP + TN)/(TP + FP + FN + TN), positive predictive value = TP/(TP + FP), and negative predictive value = TN/(FN + TN) [19, 20]. All continuous variables were expressed as mean ± SD. Categorical variables were compared with the *χ* ^2^ test and *t*-test for comparing the means of continuous variables. The *P* value 0.05 was considered significant. The chance-corrected (kappa) and chance-independent (phi) coefficients were used to obtain the exact relationship between these two tests [21].

## 3. Results

A total of 228 patient reports (*n* = 228) were involved in this retrospective cross-sectional study. These patients ages ranged from 27 years to 89 years with a mean of 59.03 ± 11.03 years. Two-thirds of the samples were male (*n* = 151) while about one-third were female (*n* = 77). Clinical characteristics of the two groups according to the results of the MPI are shown in Table 1. No significant differences were observed except for myocardial infarction (MI) and percutaneous coronary intervention (PCI). On the other hand, 26.75% of the samples revealed that they were subjected to CAG and MPI. We analyzed the MPI results and found that 78.5% of our sample was classified as having abnormal MPI results. By reviewing the means of SSS in relation to the risk factors, we found that there was a significant relationship with the risk factors (DM and HTN). Also, presenting signs and symptoms (atypical chest pain, typical chest pain, shortness of breath, and abnormal ECG) affected the means of SSS significantly.

Agreement between the MPI and CAG in detecting CAD could be assessed by many coefficients such as chance-corrected (kappa), chance-independent (phi), and Cronbach's alpha coefficients. There was a significantly fair agreement between MPI and CAG by using all the agreement coefficients (kappa = 0.237, phi = 0.310). The *P* value was 0.043 for kappa and phi. The sensitivity, specificity, and accuracy of MPI with reference to CAG were 97.8%, 20%, and 78.69%, respectively. In addition, positive predictive and negative predictive values were 78.95% and 75%. Referral bias was the cause for the increase of the sensitivity and decrease of the specificity by decreasing the number of the true negatives and false negatives. If we did not consider referral bias by including patients who were classified as negative with MPI and without referral to CAG for more than 3 months and considered them as a true negative “TN,” the sensitivity, specificity, accuracy, positive predictive value, and negative predictive value of MPI with reference to CAG would be 97.8%, 66.67%, 80.49%, 78.95%, and 96%, respectively. The sensitivity and the positive predictive value were not changed because there were no false negatives detected in those patients.

## 4. Discussion

By reviewing most of the studies in this field and to the best of our knowledge, there is no similar comparative published study. Yaghoubi and his colleagues [13] have examined the findings of CAG and MPI in cardiac syndrome X (CSX) (which is considered as one of the CAD) and found that 68.75% of MPIs showed an ischemia without a fixed lesion and transient left ventricular (TLV) dilatation. Researchers claimed that the results of the myocardial perfusion imaging were not concordant with angiographic findings, which was possibly due to the nature of this disease (nonfixed lesion). In another study, the investigator examined the value of using SPECT-MPI to detect the graft disease after a coronary artery bypass surgery (CABG), and it was claimed that “SPECT-MPI has a good sensitivity and accuracy for detecting graft disease in an unselected patient population 1 year post-CABG under optimal stress conditions” (with the presence of variation in accuracy and sensitivity of SPECT-MPI between the exercise stress test and the pharmacological stress test) [14]. On the other hand, Shelley and his colleagues [15] have attempted to compare SPECT-MPI with multislice computed tomography (MSCT), and it was found that “whenever MSCT was negative, MPI was almost negative.” In addition, a study conducted by Delcour et al. [16] included 48 patients with normal CAG and abnormal MPI who were followed up for at least 3 years from the conduction of MPI. It was found that 15 out of the studied patients had cardiovascular events, and 6 of them had coronary events (within a period of 0.5 to 8.67 years). The application of Delour's study methods on the current study was impossible as the nuclear cardiology center in KAMC was established 3 years ago. So, the retrospective follow-up of the patients who were classified as false positives for as long as 15 years is not possible. Also, patients who had a revascularization procedure showed abnormal MPI and normal CAG as MPI is more sensitive to the complex biological process that precedes restenosis earlier than CAG [12]. These results changed without intervention as the period that separates the investigation from the angioplasty changed [12]. So, for that reason it was recommended that MPI has to be before and after revascularization in order to predict the final result of perfusion [12]. Reviewing the data of those who had angioplasty procedures and had MPI and/or CAG was out of the scope of this study as we do not have access to cardiac surgery department patients results and our study was concentrating on patients who presented with a CAD clinical picture and had MPI alone or MPI and CAG as a subsequent investigation.

This significant fair agreement is not expected, but it is most probably due to referral bias, which occurs when patients with abnormal MPI results are referred for CAG at a higher rate than patients with normal MPI. These results were reviewed from a comparative perspective and it was found that a nonnegligible number was classified as a false positive, which could be justified by the high sensitivity of the MPI to minor changes of blood supply to heart muscles as long as the MPI was quantitatively investigating the richness of heart muscles with blood. On the other hand, the false negative was negligible. The very low specificity could be justified by the low number of patients with negative MPI that were subjected to CAG. This scarcity in the number of patients who did both tests was due to the invasivity of the CAG. In a comparative view of other patients' characteristics, there was a significant difference between the two groups, which was the presence or absence of a past history of MI or PCI. In the clinical scenario, patients with a past history of MI and/or PCI were more potential to develop perfusion defects.

## 5. Study Limitations

Referral bias was the main limitation in this study, as the patients who were included in this study usually were referred for CAG after observing abnormal MPI, which led to a lower number of patients matching the statistical criteria. Clinical characteristics were more liable to be affected by the recall bias from either patients or doctors; the latter was less affecting these characteristics.

## 6. Recommendation

We recommend that more studies be conducted in many tertiary centers around the country with a larger sample size of patients who had both tests.

## 7. Conclusion

In a tertiary referral center, there is a significantly fair matching between MPI and CAG results with a higher accuracy of MPI. MPI is a noninvasive diagnostic test that could be used as a gatekeeper for CAG as long as the positive predictive value is quite high compared to the negative predictive value.

## Figures and Tables

**Table 1:** Patients' characteristics according to the MPI results.

Characteristic	MPI +ve (*n* = 179)	MPI −ve (*n* = 49)	*P* value
Age	59.63 ± 10.91	56.86 ± 11.33	0.0001

Gender			
(i) Male	124 (69.3)	27 (55.1)	NS
(ii) Female	55 (30.7)	22 (44.9)	NS

Comorbidity			
(i) Diabetes	119 (66.5)	31 (63.3)	NS
(ii) Hypertension	130 (72.6)	34 (69.4)	NS
(iii) Dyslipidemia	108 (60.3)	27 (55.1)	NS
(iv) Current smoker	32 (17.88)	5 (10.2)	NS
(v) Ex-smoker	18 (10.06)	6 (12.24)	NS
(vi) Nonsmoker	2 (1.12)	1 (2.04)	NS

Presentation			
(i) Atypical chest pain	132 (73.74)	28 (57.14)	NS
(ii) Typical angina	16 (8.93)	4 (8.16)	NS
(iii) Shortness of breath	55 (30.73)	14 (28.57)	NS

Past medical history			
(i) Myocardial infarction (MI)	46 (25.7)	4 (8.2)	0.01
(ii) Coronary angiography (CAG)	39 (21.8)	7 (14.3)	NS
(iii) Percutaneous coronary intervention (PCI)	58 (32.4)	7 (14.3)	0.01
(iv) Coronary artery bypass graft (CABG)	29 (16.2)	3 (6.1)	NS

Result of resting ECG			
(i) Normal	94 (52.5)	30 (61.2)	NS
(ii) Abnormal	85 (47.5)	19 (38.8)	NS

Type of stress test			
(i) Exercise (Bruce protocol)	22 (12.3)	11 (22.4)	NS
(ii) Pharmacological (adenosine stress test)	157 (87.7)	38 (77.6)	NS

NS: not significant; ECG: electrocardiogram.

Data are numerical with percentages in the brackets or mean ± SD.

**Table 2:** Agreement between MPI and CAG.

	MPI +ve	MPI –ve
CAG +ve	45	1
CAG –ve	12	3

TP = 45; TN = 3; FP = 12; FN = 1.

The total number of patients who have been subjected to both tests is 61.
